# Embedding continuity of care into a midwifery curriculum in the Republic of Ireland: A historical context

**DOI:** 10.18332/ejm/146232

**Published:** 2022-04-12

**Authors:** Mary Curtin, Lorraine Carroll, Marcelina Szanfranska, Denise O’Brien

**Affiliations:** 1 School of Nursing, Midwifery and Health Systems, University College Dublin, Dublin, Ireland

**Keywords:** continuity, education, implementation, midwife, caseloading, academic

## Abstract

Maternity services in Ireland have historically been predominantly hospital-based and obstetric-led. Although evidence suggests midwifery-led care is safe and effective, its presence in Ireland continues to be limited in practice. An increase in the available models of maternity care for women has been recommended by the Department of Health in Ireland to promote a woman-centered approach. The latest requirement for midwifery students to have a continuity of care experience within their curriculum offers educators the opportunity to facilitate differing models of care prior to qualification as a registrant, providing an experience to explore midwifery philosophy in practice. The use of a case-loading model, adopted by a university in the East of Ireland in the final year of the program may be a successful way for students to gain midwifery skills as well as offering midwifery students the exposure to another model of care. Such experiences may also enhance their ability to drive and shape midwifery-led services in the future and also build midwifery workforce capacity in continuity of care models.

## COMMENTARY

Globally, the medicalization of birth has impacted the midwifery profession and midwifery models of care. The development of midwifery in Ireland has undoubtedly been affected by the social and cultural context peculiar to the country, and the profession has fought for autonomy since the 1918 Midwives Act (Ireland) was passed^1-4^. For most of the twentieth century, maternity services in Ireland have been predominantly hospital-based and obstetric-led^5^, alongside which midwives have struggled under a cloak of professional invisibility. Compared to other countries such as the UK, Netherlands and Australia, the medicalization of birth has become normalized in Ireland, with the focus on women’s decision making often limited. Yet, internationally, midwifery models of care continue to be proven safe and effective for a large population of women^6-9^ and are central to the improved outcomes for women and infants and achieving Sustainable Development Goals^10^.

### Access to maternity care in Ireland

The decline in autonomy and recognition of the discipline of midwifery in Ireland also translated into practice, most notably in the decline in midwifery-led care and home births. There was a significant decline in domiciliary births in Ireland in the second half of the 20th century. In 1955, there were 20665 domiciliary births out of 61622 (33.5%). By 1998, 99% of birthing women had their babies in hospitals in Ireland^11^. This trend continues today; most women give birth in maternity hospitals under the lead care of a consultant obstetrician with midwives facilitating care. In Ireland, contemporary maternity care remains fragmented, obstetric-led and continuity-of-care models are widely unavailable or inaccessible to women in the Republic of Ireland (ROI)^12^. According to the National Maternity Experience Survey (2020), consultant-led private care was offered to 55.4% of women, compared to only 15.4% who were offered the DOMINO (Domiciliary Care In and Out of hospital) scheme, with community midwifery care and homebirth accounting for 12.8% and 0.2%, respectively^13^. Furthermore, although evidence suggests the provision of midwifery led units are safe for women of low risk, only 4.1% of women in Ireland were offered midwifery-led care in a midwifery-led unit^13^. The publication of Ireland’s first National Maternity Strategy^14^ recommended the need for a strengthened role for midwifery, increased choice in models of maternity care and a woman-centered approach. Increased availability of midwifery-led models of care are intended and in progress, yet the number of midwifery-led units (MLU) in Ireland has not altered since 2004.

### Midwifery education in ROI

Midwifery in Ireland is legislated through the European Union (Article 40 of Directive 2005/36/EC). Since 2006, midwife registration in Ireland has been gained through either a four-year pre-registration Bachelor (BSc) of Midwifery program or post-registration general nurse higher diploma 18-month program (HDip Midwifery). In 2016, the Requirements and Standards for Midwife Education were updated and included, for the first time, the requirement for midwifery students to: ‘*experience the continuity of midwifery care for women, their partners and their families on at least one occasion where the student follows the woman throughout her experience of maternity care: in pregnancy, labour and birth and the postnatal period*’ through case-loading practice^15^.

The educational training of midwives in Ireland has also historically and primarily been obstetric-led and hospital-based only, with some opportunities of experience with DOMINO and community midwifery-led services. Therefore, the introduction of continuity of care experience into the midwifery curriculum is seen as another progressive step and integral to changing the future landscape of midwifery in Ireland. To date, there is limited information available to guide the implementation of this component into Irish midwifery curricula. Sharing our process may support further development of student case-loading in other pre-registration midwifery and healthcare programs in Ireland and other countries attempting to reinstate or promote midwifery models of care. Therefore, this article aims to describe the process of embedding continuity of midwifery care into the midwifery curriculum in a university in the Republic of Ireland (ROI).

### Implementation of the new policy in midwifery education in Ireland

Continuity of care is defined as midwifery care during the antenatal, intrapartum and postnatal period provided by a known midwife^16^. Women who experience continuity of midwifery care are less likely to require intrapartum analgesia or assisted birth, more likely to have a normal birth and feel in control during labor and birth, and establish breastfeeding earlier than women who have other models of care^17^.

Decisions regarding the implementation of continuity of care experiences for midwifery students were made in partnership with our affiliated clinical partner hospital to ensure the best experience for students. A caseload approach was to allow students to experience the caseload model of care and provide students with a deeper understanding of midwives’ decision-making and actions that keep women at the center of care^18^. Caseloading also provides students with an opportunity to experience and to explore midwifery philosophy in practice, and learn and build relationships with women whilst in a student capacity^19^. With adequate academic and clinical support, caseloading is student-led and student-directed rather than a ‘surface approach’ and considered an ‘additional’ aspect of the program instead of a fundamental way to gain midwifery skills^20^.

We considered the final stage of our midwifery programs, whereby students are indirectly or distantly supervised (Table 1), to be the most appropriate timeframe to include the continuity of care experience into the clinical component of the midwifery curriculum. Students practice more independently with indirect or distant supervision of an experienced/qualified midwife, thus optimizing the further development of confidence, competence, attitudes to normal midwifery practice^21^, and professional communication and skill acquisition^22^. Students gain experience of being the lead professional in caring for a small group of women throughout pregnancy, labor and birth, and the postnatal period, learning to exercise independent decision-making skills and develop skills towards autonomy.

**Table 1:** NMBI level and description of supervision (2016)

*Year*	*Level of supervision*	*Description of supervision*
One	Direct supervision	Defined as the preceptor working with the student on a continuous basis whenever care is being provided to women and their babies. The student is expected to have observed and participated in practice with the preceptor and be able to describe the care provided.
Two	Close supervision	Defined as the preceptor being present or in close proximity with the student whenever care is being provided to women and their babies. The student is expected to safely and effectively perform the skill and provide care with an underpinning rationale.
Three	Indirect supervision	Defined as the preceptor being accessible whenever the student is taking the lead in providing care to women and their babies. The student can safely and effectively perform the skill and provide care and can support care with evidence.
Four	Distant supervision	Defined as the student safely and effectively performing the skill and providing care and accepting responsibility for the provision of this care. The student is expected at all times to recognize when they need assistance from the preceptor and seek assistance in a timely manner.

### The process

**Figure 1:**
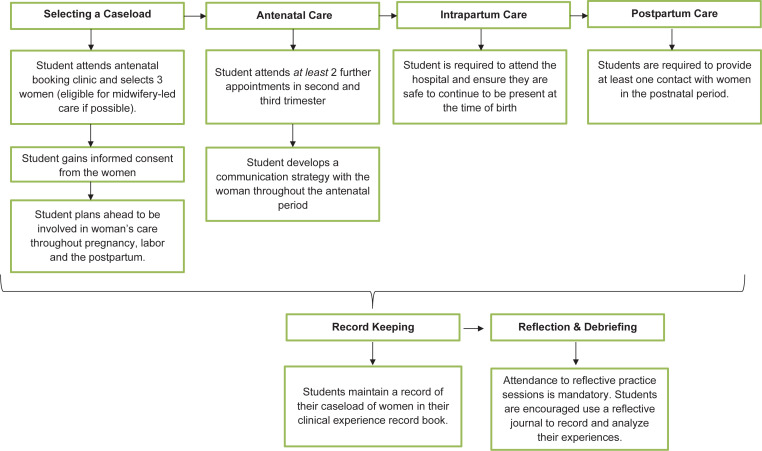
‘The Process’

The introduction of a continuity of care experience was proposed as a simple, innovative strategy to provide students with exposure to the midwifery continuity of care model. As the continuity of care model was not embedded within the clinical learning environment, the students adopted a global accompaniment with each contact point supported by the registered midwife responsible for the woman’s care at that time. Figure 1 identifies the process steps that the student must complete. As there is limited evidence guiding the assessment of continuity of care experience^23^ and it was a novel initiative in Ireland, a decision was made not to use the experience for clinical assessment purposes.

*Selecting a caseload*

Midwifery students are allocated to attend a booking antenatal clinic, and select and gain consent from three women to participate in student caseloading. Individual information leaflets on the process were designed for the woman, the student and the midwife. Women may opt out at any stage. Students are encouraged to select women who are suitable for midwifery-led care. If a woman deviates from this pathway, the student continues to be involved in the woman’s care. Students are advised to plan ahead and ensure that they will not have conflicting home and university commitments when considering the woman’s expected date of birth.

*Providing antenatal care*

Students are required to attend at least two antenatal appointments with the woman, in addition to the booking appointment, one in the second and one in the third trimester. However, it is anticipated that students will attend as many antenatal appointments as possible to build a relationship with the woman. Students are encouraged to develop a communication strategy with the woman during the antenatal period.

*Providing intrapartum care*

Students are not expected to attend the entire first stage of labor and are required to use their judgement as to when to attend the hospital to ensure they are safe to continue to be present at the time of birth. Clear instructions are also provided in the event that the student attends out of hours and/or may disrupt subsequent clinical placements.

*Providing postnatal care*

Students must provide at least one contact with women in the postnatal period.

*Reflection and debriefing*

Attendance at reflective practice days/sessions is mandatory and incorporated into the midwifery programs, therefore providing opportunities to explore and discuss issues that may evolve during clinical practice, including the continuity of care experience. Students are also encouraged to use a reflective journal to record and analyze their experiences.

*Record keeping and documentation*

**Figure 2:**
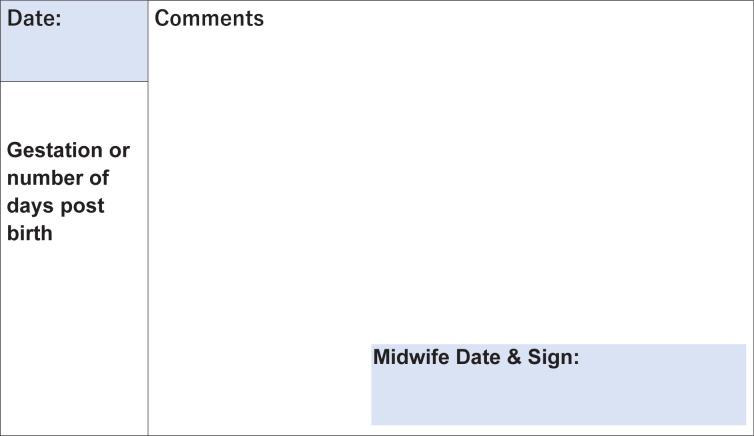
Student documentation box

Students maintain a record of their caseload of women in their clinical record of experience book (Figure 2).

## CONCLUSION

There is growing evidence and calls worldwide for strategies to strengthen the profession of midwifery. In Ireland, there is a strategic commitment within the National Maternity Strategy and midwifery education standards to make midwifery models of care more accessible to women, advocating a change in the way maternity services are delivered in Ireland. Midwifery educators are required to prepare and educate midwifery students to identify as skilled and compassionate professionals and to work in partnership with women. Midwives are expected to promote the physiological processes of pregnancy, birth, postpartum and the early weeks of life. This initiative is one strategy to help strengthen the profession of midwifery in Ireland. Providing students with similar experiences can also enhance their ability to drive and shape midwifery-led services in the future and continue to build midwifery workforce capacity in the continuity of care models, thus contributing to safe and high-quality maternity care and efficient use of resources.

Internationally, there is evidence of the benefits and challenges of integrating continuity of care models. The main driver for continuity of care models is the evidence that has emerged around improved relationships, enhanced communication and overall greater satisfaction of care^17,24,25^ whilst benefits for midwives include enhanced autonomy^26^. However, evidence from New Zealand, known for its world-leading model of continuity of care, highlights burnout and work-life balance as concerns for caseloading midwives^27^. Yet, a qualitative study with 13 midwives in Denmark suggested the benefits of caseloading outweigh the disadvantages^28^.

The approach to the inclusion of this mandated clinical practice-based experience may vary across institutions. Although welcomed, there are also significant challenges as ongoing issues exist in terms of accessibility and equality of midwifery-led services and continuity of care from a national perspective. A research study to evaluate midwifery students’ views and experiences of embedding continuity of care practice experience into midwifery curricula in a large urban university in the East of Ireland is in progress to gather information from the student perspective. Understanding the perspectives of women and midwives supporting students to achieve continuity of care requirements from an Irish perspective also requires further research to ascertain the benefits and disadvantages in the Irish context.

## Data Availability

Data sharing is not applicable to this article as no new data were created.
